# Management of Asymptomatic Severe Aortic Stenosis: Current Evidence and Future Directions

**DOI:** 10.3390/jcm14217549

**Published:** 2025-10-24

**Authors:** Giulia Laterra, Federica Agnello, Orazio Strazzieri, Claudia Reddavid, Lorenzo Scalia, Salvatore Ingala, Simona Guarino, Chiara Barbera, Maria Daniela Russo, Giuliano Costa, Marco Barbanti

**Affiliations:** 1 Faculty of Medicine and Surgery, Università degli Studi di Enna “Kore”, Piazza dell’Università, 94100 Enna, Italy; giulia.laterra@unikore.it (G.L.); federicagiuseppa.agnello@gmail.com (F.A.); 2 Division of Cardiology, Ospedale Umberto I, ASP 4 di Enna, 94100 Enna, Italy; o.strazzieri@gmail.com (O.S.); claudiareddavid@gmail.com (C.R.); lorenzoscalia1993@gmail.com (L.S.); ingalasalvatore@gmail.com (S.I.); simonaguarino@hotmail.it (S.G.); chiara.barbera.me@gmail.com (C.B.); danielarusso28@hotmail.it (M.D.R.); 3 Division of Cardiology, A.O.U. Policlinico “G. Rodolico San Marco”, 95123 Catania, Italy; giulianocosta90@gmail.com

**Keywords:** aortic valve replacement, Asymptomatic Severe Aortic Stenosis, transcatheter aortic valve replacement

## Abstract

Systematic Severe aortic stenosis (AS) is a progressive disease and one of the most common valvular heart diseases in developed countries. The management of asymptomatic severe AS remains challenging and requires a tailored, patient-specific approach. Optimal timing of intervention in asymptomatic patients continues to be a matter of ongoing debate. In individuals with severe AS and reduced left ventricular ejection fraction (LVEF), both the ESC/EACTS and ACC/AHA guidelines recommend aortic valve replacement (AVR), regardless of symptom status. In contrast, for patients with preserved LVEF (≥50%), the decision to proceed with AVR must be individualized and based on a comprehensive risk assessment. Risk stratification plays a central role in guiding early intervention strategies and should incorporate clinical findings, echocardiographic parameters, biomarkers, and advanced imaging techniques such as cardiac magnetic resonance and computed tomography. Recent randomized controlled trials have yielded mixed results regarding the mortality benefit of early AVR but have consistently demonstrated a reduction in heart failure-related hospitalizations. Timely intervention in carefully selected high-risk patients may improve long-term outcomes, while avoiding unnecessary procedures in lower-risk individuals remains equally important.

## 1. Introduction

Severe aortic stenosis (AS) is a progressive disease and one of the most common valvular disorders in developed countries, affecting approximately 5% of patients over 65 years, with a poor prognosis if not treated [1]. In Europe and North America, the most common causes of aortic stenosis include calcific degeneration of a tricuspid valve or a bicuspid aortic valve (BAV) with superimposed calcification. In up to 50% of patients with BAV, ascending aorta dilatation (also known as BAV aortopathy) is observed [2,3]. Less commonly, rheumatic valve disease and congenital aortic stenosis due to a unicuspid valve may also be observed [2,3]. Aortic valve replacement (AVR) remains the only strategy shown to improve survival, as medical therapy neither corrects nor slows the progression of the disease [4,5,6,7]. However, ongoing studies are exploring pharmacological approaches, such as reactivation of oxidized sGC with ataciguat, which, in a 6-month trial, slowed the progression of aortic valve calcification and showed a tendency to reduce the progression of valvular and ventricular dysfunction in patients with moderate fibrocalcific aortic valve stenosis [8]. Even in patients classified as “asymptomatic,” subtle reductions in exercise tolerance, fatigue, and psychological distress may precede overt symptoms and significantly affect quality of life (QoL). Recent evidence shows that while AVR leads to a marked improvement in QoL over time, a subset of patients, particularly those with comorbidities such as peripheral artery disease or paravalvular leak, experience persistently impaired health-related QoL, underscoring the importance of incorporating patient-reported outcomes in clinical assessment and decision-making [9].

## 2. Current Guidelines and Indications for Aortic Valve Replacement

Current guidelines recommend AVR, either surgical (SAVR) or transcatheter (TAVR) with varying levels of indication based on symptom status and echocardiographic assessment of the severity of AS [10,11,12]. Recently, Généreux et al. introduced a clinical classification for AS that stratifies patients into three groups based on signs and symptoms: stable valve syndrome, progressive valve syndrome (PVS), and acute valve syndrome (AVS). These categories correspond, respectively, to asymptomatic patients, those with mild and progressively worsening symptoms, and those presenting with acute, advanced, or severe symptoms. This framework offers a practical tool to guide prognosis and treatment decisions [13].

Symptomatic severe high-gradient AS represents a well-established indication for intervention (Class I, Level B), regardless of left ventricular ejection fraction (LVEF) as prognosis without intervention is poor. Symptomatic paradoxical low-flow, low-gradient AS requires further diagnostic evaluation using tools such as dobutamine stress echocardiography (DSE) and/or cardiac computed tomography (CCT). DSE provides information on the changes in mean gradient, peak velocity, aortic valve area (AVA) and flow reserve. Absence of flow reserve, defined as failure to increase stroke volume by more than 20%, is associated with higher surgical mortality and worse long-term outcomes. CCT-based aortic valve calcium scoring are recommended to confirm the severity of the stenosis and guide referral for intervention (Table 1) [10,11,12,14,15].

The management of asymptomatic severe AS is more complex and requires considerations and a tailored approach. First, it is essential to confirm that the patient is truly asymptomatic; patients may subconsciously adapt their lifestyle and thus underestimate or underreport symptoms. Moreover, interpreting dyspnea as a cardiac symptom is often complex in these patients and symptom assessment can be particularly limited in very elderly, frail patients with multiple comorbidities. Indeed, 20% of patients with asymptomatic AS are unable to perform a stress test due to reduced mobility or exercise tolerance. However, patients with positive stress test had a 7.6-fold increased risk of developing symptoms or sudden death at follow-up [16,17,18]. Multivariable analysis in the study by Das et al. identified exercise-induced limiting symptoms as the most powerful independent predictor of symptom appearance within 12 months [17]. Therefore, stress testing is recommended for risk stratification, in order to unmask symptoms and assess haemodynamic intolerance, such as a drop in blood pressure during exertion. Exercise stress echocardiography and cardiopulmonary exercise testing provide complementary information, assessing transvalvular gradient changes during exertion and aiding in the differential diagnosis of symptoms, respectively [15] In approximately one-third of asymptomatic patients, stress testing can unmask latent symptoms, allowing these patients to be managed similarly to those with overt symptoms. However, the predictive value of the test is lower in older patients and in those with comorbidities, such as diabetes mellitus, which may mask symptoms. Moreover, AVR should be considered in asymptomatic patients with severe AS and a sustained fall in BP (>20 mmHg) during exercise testing (Class IIa, Level of Evidence C) [15].

Current 2025 ESC/EACTS and 2020 ACC/AHA guidelines, recommend AVR in patients with LVEF < 50% without another cause (Class I, Level of Evidence B) [15]. The ACC/AHA guidelines recommend considering AVR in those with an LVEF < 60% and evidence of progressive decline in contractile function demonstrated by imaging (Class II, Level of Evidence B) [10]. In contrast, the latest 2025 ESC/EACTS guidelines recommend AVR in patients with an LVEF < 50% in the absence of other causes (Class I, Level of Evidence A) [14]. The ESC/EACTS recommendation is based on the study by Bohbot, which compared three LVEF groups: <55%, 55–59%, and ≥60%. The study showed that patients with LVEF <55% had significantly higher mortality compared to those with LVEF ≥ 60%, regardless of initial conservative or surgical management with an adjusted hazard ratio of 2.44 (95% CI: 1.51–3.94; *p* < 0.001). In contrast, patients with LVEF between 55% and 59% had a similar prognosis to those with LVEF ≥ 60%. Moreover, among patients with LVEF < 55%, initial conservative management was associated with increased mortality compared to early surgery [19].

In asymptomatic patients with severe AS and LVEF ≥ 50%, the decision to proceed with aortic valve intervention must be carefully individualized. A stress test should be considered to unmask symptoms and assess for an abnormal blood pressure response. However, even with a normal stress test, compared with previous guidelines, the new ones stated that AVR should be considered in patients with severe AS and low procedural risk (Class IIa, Level of Evidence A) [15]

**Figure 1:**
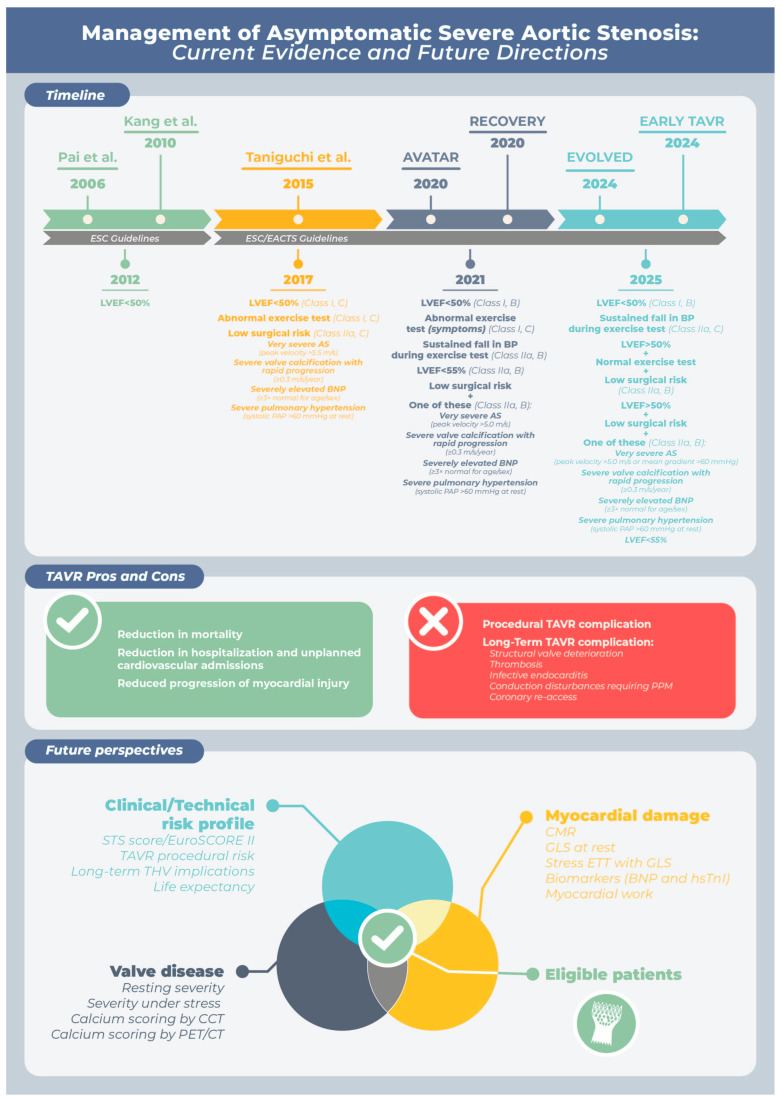
From the top, the figure illustrates the progressive shift in clinical decision-making over time. It begins with key studies comparing early aortic valve replacement (AVR) with a conservative surveillance strategy, followed by the evolution of ESC guideline recommendations for asymptomatic aortic stenosis (AS), highlighting changes in classes of indication from 2012 to 2025. The next section summarizes the main advantages and limitations to consider when evaluating early AVR in asymptomatic patients. At the bottom, the figure presents the future perspective, which should focus on identifying patients who are most likely to benefit from early intervention. This requires the integration of multiple parameters: valve severity assessed both at rest and during stress (using modalities such as cardiac CT and PET), myocardial injury detected through advanced imaging techniques such as cardiac MRI and strain imaging (including GLS during stress echocardiography), relevant circulating biomarkers, and the patient’s clinical and technical profile—including frailty, procedural risk (e.g., EuroSCORE II, STS score), procedural TAVR complications, and long-term transcatheter heart valve (THV) considerations. Long-term complications of TAVR include structural valve deterioration (SVD) and non-structural valve dysfunction (NSVD), as well as other causes of valve failure such as thrombosis and infective endocarditis. Additionally, the risk of conduction disturbances requiring permanent pacemaker implantation and challenges related to future coronary re-access should be carefully considered. This multimodal approach may help guide more personalized, evidence-based decision-making in the management of asymptomatic severe AS. AVR = aortic valve replacement; BNP = B-type Natriuretic Peptide; BP = Blood pressure; CCT = cardiac computed tomography; CMR = cardiac magnetic resonance; EACTS = European Association for Cardio-Thoracic Surgery; ETT = transthoracic Echocardiogram; ESC = European Society of Cardiology; EUROSCORE II = European System for Cardiac Operative Risk Evaluation II; GLS = global longitudinal strain; hs-TnI = high-sensitivity Troponin I LVEF = left ventricular ejection fraction; PPI = permanent pacemaker implantation; STS score = Society of Thoracic Surgeons score; TAVR = transcatheter aortic valve replacement; THV = Transcatheter Heart Valve; Vmax = Peak Velocity. The studies shown in the figure are: Pai et al. [59], Taniguchi et al. [60], Kang et al. [61], AVATAR [63], RECOVERY [64], EVOLVED [65], and EARLY TAVR [66].

Moreover, although clinical surveillance (CS) remains a reasonable approach, AVR should be considered, particularly in patients with low procedural risk, when specific high-risk features indicative of disease progression or adverse outcomes are present. These include very severe AS (mean gradient ≥ 60 mmHg or Vmax > 5.0 m/s), severe valve calcification with rapid progression of transvalvular velocity (≥0.3 m/s/year), markedly elevated natriuretic peptide levels (BNP or NT-proBNP >3 times the age- and sex-adjusted normal values, confirmed and without alternative explanation), or a reduction in LVEF to below 55% in the absence of other identifiable causes. In such patients, early intervention may improve long-term outcomes and reduce the risk of sudden symptom onset or cardiovascular events (Class IIa, Level of Evidence B) [15] (Figure 1).

The 2025 ESC/EACTS guidelines introduce several important updates compared to the 2021 version, particularly in the management of asymptomatic severe AS. While a LVEF < 50% remains a Class I indication for AVR, the new guidelines recommend earlier intervention in asymptomatic patients with preserved LVEF when specific high-risk features are present, such as a peak aortic jet velocity > 5.0 m/s, rapid progression of stenosis (increase in Vmax ≥ 0.3 m/s/year), markedly elevated natriuretic peptides, or an abnormal blood pressure response to exercise. Risk stratification has become more nuanced, allowing for tailored decision-making based on individual patient profiles. Advanced imaging modalities, especially computed tomography (CT) for aortic valve calcium scoring, are now strongly emphasized to support diagnosis in patients with low-gradient or discordant echocardiographic findings. Furthermore, a notable change in the age threshold for recommending TAVR has been introduced: the preferred cutoff has shifted from 75 to 70 years in patients with tricuspid aortic valves and suitable anatomy. Together, these changes reflect a broader move toward personalized, earlier, and more evidence-based intervention strategies in asymptomatic AS [20,21]. In conclusion, the ESC/EACTS 2025 and ACC/AHA 2020 guidelines share several core principles regarding the management of AS, yet they differ in some key aspects. Both recommend AVR in symptomatic severe AS and in asymptomatic patients with LVEF < 50% (ESC: Class I, Level of Evidence A; ACC/AHA: Class I, Level of Evidence B). However, the ACC/AHA guidelines are more permissive, suggesting consideration of AVR at LVEF < 60% if there is evidence of progressive decline in systolic function (Class IIa). The ESC guidelines take a more conservative stance, reserving intervention for LVEF < 50%, supported by recent evidence showing increased mortality below this threshold. In asymptomatic patients with preserved LVEF, both guidelines now endorse consideration of AVR in the presence of high-risk features, such as very severe AS, rapid progression, elevated BNP, or exercise-induced blood pressure drop, particularly in patients with low procedural risk.

## 3. Predictors of Progression

The progression of AS, along with age, chronic heart failure, stenosis severity, and the degree of valve calcification, is closely associated with clinical outcomes. Several factors have been identified as predictors of rapid AS progression, including smoking, diabetes mellitus, hypertension, chronic kidney disease, dyslipidemia, coronary artery disease, and male sex [22]. Approximately 70% of sudden deaths in patients with asymptomatic severe AS occur without any preceding symptoms [23]. This underscores the importance of identifying individuals at higher risk of experiencing adverse cardiac events. Various echocardiographic parameters have been recognized as potential risk markers, such as a peak aortic jet velocity (Vmax) greater than 5 m/s [24,25], a progression rate of peak transvalvular velocity exceeding 0.3 m/s per year, a stroke volume index (SVi) below 35 mL/m^2^ [26], reduced LVEF [8,27,28,29,30], left atrial enlargement [31,32], left ventricular hypertrophy, pulmonary hypertension, increased valvuloarterial impedance (greater than 4.5), and reduced (<−15%) global longitudinal strain (GLS). Additionally, the presence of inappropriate left ventricular mass, defined as more than 110% of the predicted value, has also been associated with worse outcomes.

## 4. Advanced Imaging for Risk Stratification

Many of these echocardiographic indicators identify patients with established myocardial damage, such as reduced stroke volume, or suggest early myocardial injury even when LVEF remains preserved. This underscores the critical importance of assessing the extent of extra-valvular cardiac involvement, which may play a key role in identifying patients at increased risk of clinical outcomes. Initially, left ventricular hypertrophy represents a compensatory response to the increased afterload caused by aortic valve narrowing, aiming to maintain cardiac performance. However, this adaptation comes at the cost of progressive myocyte death and myocardial fibrosis, which ultimately underlie the transition from a compensated to a decompensated state [33,34,35]. Impairment in LVEF is often a late manifestation that may not be reversible.

Based on this premise, there is a clear need to identify early markers of both myocardial and valvular involvement before irreversible damage occurs, damage that can negatively impact outcomes even after TAVR. Myocardial fibrosis progresses rapidly and serves as a strong, independent predictor of incident heart failure, as well as all-cause and cardiovascular mortality in patients with AS. In this context, new and advanced imaging techniques play a crucial role.

### 4.1. Echocardiographic Parameters

Echocardiography remains the cornerstone for both initial evaluation and serial monitoring of asymptomatic patients with AS. Several echocardiographic markers have been shown to predict adverse outcomes, including Vmax, degree of valve calcification, progression rate, AVA, and LVEF. Among these, Vmax is a strong prognostic indicator: patients with very severe AS (Vmax > 5 m/s) have significantly higher all-cause and cardiovascular mortality, even in the absence of symptoms or following AVR. The HAVEC registry highlighted a 4-year survival of only 65–78% in patients with Vmax > 5 m/s, supporting consideration for earlier intervention. Rapid progression (Vmax increase > 0.3 m/s/year) and moderate/severe valve calcification are also associated with a high (79%) 2-year risk of symptom onset or death. Multivariable analyses identified both reduced LVEF and elevated Vmax as independent predictors of mortality. Optimal cutoff values for risk stratification were 4.7 m/s for Vmax. An AVA < 0.8 cm^2^ was also associated with markedly increased mortality risk. These parameters are critical for identifying high-risk patients who may benefit from closer surveillance or early AVR, even in the absence of symptoms [36,37,38,39].

### 4.2. Myocardial Fibrosis and Cardiac Magnetic Resonance

Cardiac magnetic resonance imaging (CMR) with late gadolinium enhancement (LGE) is employed to detect areas of myocardial fibrosis. Unlike the fibrosis associated with coronary artery disease, the interstitial fibrosis related to aortic stenosis is diffusely distributed, reflecting the more uniform and progressive nature of the condition [8]. The role of CMR in this setting was investigated in the EVOLVED study, a multicenter, prospective, randomized, open-label trial. Patients with asymptomatic severe AS were initially screened for adverse left ventricular remodeling by measuring plasma cardiac high-sensitivity troponin I (hsTnI), with a threshold of ≥6 ng/L, or by the presence of left ventricular hypertrophy on electrocardiography. Those with hsTnI levels above this threshold or evidence of hypertrophy were referred for CMR. Patients in whom midwall LGE was detected were then randomized to receive either AVR or a CS. The EVOLVED trial showed that early AVR did not have a significant effect on the combined primary endpoint of all-cause mortality or unplanned aortic stenosis-related hospitalizations in asymptomatic patients with severe AS and myocardial fibrosis, compared to guideline-directed conservative management. However, it should be noted that the median time to intervention in the early intervention group may have been too long, potentially influencing the results. Additionally, the study showed that patients receiving a strategy of CS experienced a higher burden of heart failure symptoms at 12 months. The reduction in unplanned hospitalizations related to AS remains an important therapeutic goal for many elderly patients, especially considering the low procedural risk associated with early AVR [40]. However, in the EVOLVED trial, early AVR included both SAVR and TAVR. Combining these modalities under a single category represents a study limitation.

### 4.3. Valve Calcification and Computed Tomography

Severe aortic valve calcification is also strongly associated with increased all-cause mortality, and patients with more advanced disease at baseline tend to experience a more rapid progression of calcification. This progression of aortic valve narrowing appears to be driven by a self-perpetuating cycle of calcium accumulation [41,42,43]. To enhance diagnostic accuracy and risk stratification, CCT-based aortic valve calcium scoring shows promise as an alternative method for assessing the severity of aortic stenosis, demonstrating high intra- and interobserver reproducibility compared to echocardiographic evaluation. Importantly, sex-specific threshold values should be applied, with lower cutoffs in women (>1200 AU) compared to men (>2000 AU), to more accurately identify severe aortic stenosis and better predict clinical outcomes [44,45]. An integrated positron emission tomography (PET)/CT approach has been used for the assessment of aortic stenosis. The PET tracer 18F-fluoride has a high affinity for newly developing microcalcifications and functions as a marker of ongoing calcification [18,46].

### 4.4. Global Longitudinal Strain

In addition, GLS assessed by two-dimensional (2D) speckle-tracking analysis provides useful prognostic information in asymptomatic patients with severe AS. Nagata et al. demonstrated that 2DGLS, 3DGLS and 3D global radial strain (3DGRS) were significantly reduced in patients who subsequently experienced major adverse cardiovascular events (MACE) compared to those who did not, and these parameters effectively identified a subgroup of patients at higher risk [47]. Patients with an absolute GLS value below 14.7% have a 2.5-fold increased risk of mortality [48]. Lancellotti et al. and Yingchoncharoen et al. also demonstrated that 2DGLS may play a role in identifying patients at high risk of cardiovascular events [49,50].

### 4.5. Myocardial Work

Conventional indices of left ventricular systolic function, such as LVEF and GLS, are based on the assessment of myocardial fiber shortening and do not account for LV afterload, which is particularly relevant in patients with severe AS [51,52,53]. Russell et al. developed a noninvasive method to calculate LV myocardial work indices by integrating echocardiographic strain data with systolic blood pressure to derive pressure–strain loops [51,52,54]. These indices have shown strong correlations with invasively measured myocardial performance and oxygen consumption assessed by positron emission tomography. Among them, global constructive work (GCW) and global work index (GWI), which estimate the degree of LV adaptation to increased afterload, have demonstrated an independent association with NYHA functional class III or IV heart failure symptoms, and may offer additional insights into myocardial function beyond conventional, afterload-dependent echocardiographic parameters. Moreover, global work efficiency (GWE) has emerged as a key marker, reflecting the proportion of effective myocardial work relative to total work performed, and thereby integrating both mechanical function and energetic efficiency. In asymptomatic patients with severe AS, reductions in GCW, GWI, or GWE may indicate early subclinical myocardial dysfunction despite preserved LVEF, identifying patients at higher risk of progression [54]. Recent data from the EffecTAVI registry showed that a GWE ≤ 92% at 30 days after TAVR strongly predicted adverse clinical outcomes at 1-year follow-up. Additionally, significant reductions in GWI, GCW, and GWW were observed after TAVI, while GWE improved only in patients without new-onset dyssynchrony [55]. By incorporating afterload into their calculation, myocardial work indices may improve the assessment and longitudinal follow-up of LV systolic function and help refine the timing of aortic valve intervention, before irreversible ventricular damage occurs [54].

### 4.6. Biomarkers of Myocardial Injury

Biomarkers such as N-terminal pro–B-type natriuretic peptide (NT-proBNP), the active hormone B-type natriuretic peptide (BNP), and hsTnI appear to play a role in the early identification of myocardial damage, and elevated levels are associated with a higher risk of adverse events, including death and AVR, at a mean follow-up of 1.5 years [23,56,57]. Furthermore, BNP and NT-pro BNP serve as predictors for symptom development and may be useful in selecting patients for early TAVR [56]. In the biomarker substudy of the EARLY TAVR trial, baseline levels of NT-proBNP and hs-cTnT were associated with higher event rates, but did not significantly modify the relative benefit of early TAVR in asymptomatic patients with severe high-gradient aortic stenosis. The study initially hypothesized that patients with higher levels of cardiac biomarkers (NT-proBNP and hs-cTnT), indicative of more advanced myocardial damage, would derive the greatest benefit from early TAVR due to prompt unloading of the left ventricle. Surprisingly, the results contradicted this hypothesis. Early TAVR showed a consistent clinical benefit across all biomarker levels, and the relative benefit, measured by hazard ratios (HR), was numerically greater in patients with lower or normal biomarker levels (e.g., HR = 0.00 in the lowest NT-proBNP tertile vs. HR = 0.36 in the highest). However, when assessing absolute benefit, measured by the number needed to treat (NNT), the results were mixed. Patients with higher biomarker levels, who had higher baseline risk, showed greater absolute benefit from early TAVR (NNT = 11 in the highest NT-proBNP tertile) compared to those with lower biomarker levels (NNT = 32) [58]. These findings reinforce the primary results of the EARLY TAVR trial and suggest that single measurements of these biomarkers have limited value in guiding optimal TAVR timing in asymptomatic patients. In contrast, the EVoLVeD trial adopted a biomarker-guided approach by enrolling only patients with detectable hs-cTnT or electrocardiographic evidence of left ventricular hypertrophy and confirmed myocardial fibrosis on imaging. While this design allowed for the selection of a higher-risk cohort, it did not permit evaluation of treatment effects across the full range of biomarker concentrations. Therefore, although both trials highlight the prognostic value of NT-proBNP and hs-cTnT in asymptomatic severe AS, current evidence suggests that these biomarkers alone are insufficient to guide clinical decision-making regarding the timing of TAVR.

## 5. Early Intervention vs. Clinical Surveillance: Evidence from Observational Studies and Randomized Clinical Trials

### 5.1. Observational Studies

Observational studies have shown that the majority of patients with asymptomatic severe aortic stenosis develop symptoms within 1–2 years of diagnosis, with annual mortality risk of 1–2%.

As early as 2006, Pai et al. [59], in a retrospective observational study, reported a significant survival difference between patients who underwent early AVR and those managed with clinical CS. Survival at 1, 2, and 5 years in the CS group was 67%, 56%, and 38%, respectively, compared to 94%, 93%, and 90% in the AVR group (*p* < 0.0001) [59]. Similarly, Taniguchi [60] and Kang et al. [61], using propensity score-matched analyses, demonstrated a significant survival benefit with early AVR compared to CS. Taniguchi et al. reported a lower cumulative 5-year incidence of all-cause death in the early AVR group (15.4% vs. 26.4%; *p* = 0.009) [60]; Kang et al. found a markedly reduced all-cause mortality (HR: 0.14; 95% CI: 0.03–0.60; *p* = 0.008), with 6-year overall and cardiac mortality-free survival rates of 98% and 100% in the early AVR group versus 68% and 76% in the CS group (both *p* < 0.001) [61]. Similarly, Brown et al. demonstrated that, among patients with asymptomatic severe AS, the omission of AVR was the most significant risk factor for late mortality (HR: 3.53; *p* < 0.001). Moreover, operative mortality also appears to be lower with early AVR compared to late AVR (1.9% vs. 2.8%) [62].

Beyond survival, Taniguchi et al. also assessed symptom onset and heart failure-related hospitalizations, reporting that early AVR was associated with significantly lower 5-year incidences of both new symptom development (3.2% vs. 46.3%; *p* < 0.001) and heart failure hospitalization (3.8% vs. 19.9%; *p* < 0.001) [60]. (Table 2)

### 5.2. Randomized Clinical Trials

There are four randomized controlled trials (RCT): AVATAR and RECOVERY, which evaluated SAVR; EVoLVeD, which included both TAVR and SAVR; and EARLY TAVR, which specifically assessed TAVR as a treatment strategy (Figure 1) (Table 2).

The AVATAR (Aortic Valve Replacement Versus Conservative Treatment in Asymptomatic Severe Aortic Stenosis) trial was an investigator-initiated, prospective, multicenter, randomized controlled trial designed to compare the safety and efficacy of early SARV versus a CS strategy in truly asymptomatic patients with severe AS and LVEF ≥ 50% [63]. To confirm the asymptomatic status, all patients underwent an exercise test and were able to achieve 85% of the maximum predicted heart rate without symptoms. A total of 157 patients were enrolled, with potential unrecognized symptoms excluded by performing an exercise stress test. A primary outcome event, defined as a composite of all-cause mortality or major adverse cardiovascular events, including acute myocardial infarction, stroke, and unplanned heart failure hospitalization requiring intravenous diuretics or inotropes, occurred in 23% of patients in the early SAVR group and in 46.8% of those in the clinical surveillance group (HR for early SAVR vs. CS: 0.42; 95%, CI: 0.24–0.73; *p* = 0.002). Kaplan–Meier estimates of the individual endpoints of all-cause death and HF hospitalization were significantly lower in the early SAVR compared with the CS group (HR 0.44; 95% CI 0.23–0.85, *p* = 0.012, for all-cause death and HR 0.21; 95% CI 0.06–0.73, *p* = 0.007, for HF hospitalizations). Taking into consideration that the trial was underpowered and ended prematurely, patients randomized to early surgery had a significantly lower incidence of the primary composite endpoint (15.2% vs. 34.7%; HR: 0.46; 95% CI: 0.23–0.90; *p* = 0.02). No differences were found in overall mortality. Approximately 49.4% of patients in the conservative group eventually underwent AVR, most commonly due to onset of symptoms (51.4%). The annual rate of sudden cardiac death in asymptomatic patients in the conservative group was 1.48% vs. 1% in the early surgery group.

The RECOVERY (Randomized Comparison of Early Surgery versus Conventional Treatment in Very Severe Aortic Stenosis) trial was a multicenter, randomized, open-label trial that compared early SAVR with a CS strategy [64]. In this trial, 145 patients with LVEF ≥ 50% and very severe aortic stenosis, defined as an AVA < 0.75 cm^2^, peak velocity (Vmax) > 4.5 m/s, or mean gradient > 50 mmHg, were randomized. To confirm asymptomatic status, exercise testing was performed selectively, and patients with a positive test were excluded. The primary endpoint, defined as operative mortality or death from cardiovascular causes during follow-up, occurred in 1% of patients in the early SAVR group versus 15% in the CS group (OR: 0.09; 95% CI: 0.01–0.67). During follow-up, 74% of patients in the CS group eventually underwent SAVR after a median of 23 months. Overall mortality was significantly lower in the early surgery group (7% vs. 21%; HR: 0.33; 95% CI: 0.12–0.90), while the annual rate of sudden cardiac death in the CS group was 1% at 4 years. Both overall and cardiovascular mortality increased markedly between 6 and 8 years, suggesting less intensive surveillance in later years. Additionally, the incidence of hospitalization for heart failure was lower in the early SAVR group, with no reported cases compared to 11% in the CS group (OR: 0.05; 95% CI: 0.00–1.05).

The EVoLVeD (Early Valve Replacement Guided by Biomarkers of Left Ventricular Decompensation in Asymptomatic Patients with Severe Aortic Stenosis) trial was designed to investigate whether early aortic valve intervention can improve clinical outcomes in patients with asymptomatic severe aortic stenosis and evidence of myocardial fibrosis [65]. The design of this randomized trial has been previously described. The primary endpoint, defined as a composite of all-cause mortality or unplanned aortic stenosis–related hospitalization during the follow-up period, occurred in 18% of patients in the early intervention group and 23% in the guideline-directed conservative management group. The difference between groups was not statistically significant (HR 0.79; 95% CI: 0.44–1.43; *p* = 0.44). However, at 12 months, patients in the conservative management group showed a higher burden of heart failure symptoms, a finding not observed in those who underwent early aortic valve intervention. This was associated with a statistically significant reduction in unplanned aortic stenosis–related hospitalizations in the early intervention group (HR 0.37; 95% CI: 0.16–0.88).

The EARLY TAVR (Evaluation of TAVR Compared to Surveillance for Patients with Asymptomatic Severe Aortic Stenosis) trial was a prospective, multicenter, open-label, randomized, controlled trial in which TAVR with SAPIEN 3 or SAPIEN 3 Ultra (Edwards Lifesciences, Irvine, CA, USA) valves was compared with CS among patients with asymptomatic severe AS [66]. Patients included were ≥65 years, with severe AS and LVEF ≥ 50%, and suitable anatomy for transfemoral TAVR. Exercise testing was performed in 90.6% of patients to confirm asymptomatic status.

The composite endpoint of death, stroke, or unplanned hospitalization for cardiovascular causes occurred in 26.8% of patients in the early TAVR group compared to 45.3% in the clinical surveillance group, corresponding to a significant risk reduction (HR 0.50; 95% CI, 0.40–0.63). Overall mortality was similar between groups (8.4% vs. 9.2%), and the annual rate of sudden cardiac death was approximately 0.4% in the TAVR group and 0.3% in the CS group. In the CS group, 23% of patients underwent TAVR within 6 months, and 87% received TAVR after a median of 11 months, most commonly due to onset of symptoms. The 30-day mortality after TAVR was 0.9%.

The EASY-AS (Early Valve Replacement in Severe ASYmptomatic Aortic Stenosis) trial is an ongoing randomized controlled trial designed to evaluate whether early AVR (surgical or transcatheter) reduces cardiovascular mortality and heart failure hospitalizations, and improves other important outcomes [67].

## 6. Reconsidering the Risk–Benefit Balance in the Modern Era: Between Light and Shadow

### 6.1. Mortality Outcomes in Early AVR Versus Clinical Surveillance

Mortality remains a key endpoint in randomized trials comparing early AVR versus CS in asymptomatic patients with severe AS, yet findings have been notably heterogeneous. The RECOVERY and AVATAR trials demonstrated a significant reduction in all-cause mortality with early surgical AVR: RECOVERY: HR 0.33 (95% CI, 0.12–0.90); AVATAR: HR 0.44 (95% CI, 0.23–0.85). In contrast, the EARLY TAVR and EVOLVED trials did not demonstrate a statistically significant survival benefit. Specifically, in EARLY TAVR, all-cause mortality occurred in 8.4% of patients in the early TAVR group versus 9.2% in the CS group (HR 0.93; 95% CI, 0.60–1.44), while in EVOLVED, the hazard ratio for mortality was 1.22 (95% CI, 0.59–2.51). This discrepancy among these four RCT may be attributed to two main factors. First, RECOVERY and AVATAR enrolled relatively younger patients (mean age 64.5–67.0 years) with severe or critical AS, a population more likely to derive a mortality benefit from early intervention. In contrast, EARLY TAVR and EVOLVED included older cohorts (mean age 73.4–75.8 years), in whom competing comorbidities and reduced life expectancy may dilute the potential survival advantage of early AVR. Second, the degree of stenosis severity at enrollment appears to have differed. Patients in EARLY TAVR and EVOLVED had, on average, less advanced AS, as reflected by higher AVA, lower Vmax, and lower mean transvalvular gradients compared to those in RECOVERY and AVATAR. These differences in baseline disease severity likely influenced outcomes and may partially explain the absence of a mortality benefit in the more recent trials (Table 3). Moreover, according to Genereux et al. [66], the differences in mortality outcomes across trials may be less related to the time from symptom onset to intervention in the CS groups. In particular, the median time from symptom recognition to AVR was significantly longer in the surgical trials, 4.0 months in AVATAR and 3.3 months in EVOLVED, compared to 32 days in EARLY TAVR. This prolonged exposure to symptomatic severe AS in the surgical trials may have contributed to higher mortality rates while awaiting AVR [68]. Loganath et al. suggest that the lack of statistical significance for mortality observed in the EVOLVED trial may instead be attributed to the relatively long median time to early intervention in the treatment group [69] Furthermore, the results of the EVOLVED trial may have been influenced by the lack of a specific fibrosis cutoff value to identify high-risk patients. According to a study by Lee et al. [70], patients with an extracellular volume fraction (ECV%) greater than 28.6% showed significantly worse outcomes. Additionally, patients who experienced events had higher median ECV% values compared to those without events (28.2% vs. 26.3%; *p* = 0.003) [70]. This suggests that quantification of myocardial fibrosis could play a key role in risk stratification, potentially affecting the interpretation of trial results where fibrosis cutoffs were not established. Similarly, Everett et al. observed that an indexed ECV% greater than 22.5 mL/m^2^ and the presence of mid-wall LGE were associated with increased all-cause mortality; however, a specific cutoff value for ECV% was not defined [71]. Although the composite endpoint of death, stroke, or unplanned cardiovascular hospitalization in EARLY TAVR favored early intervention, the result was primarily driven by a reduction in unplanned hospitalizations (HR 0.43; 95% CI, 0.33–0.55). Mortality did not differ substantially between the two groups at any time. Importantly, AVR in the CS group was only considered an unplanned hospitalization if it occurred within the first 6 months, potentially biasing the composite outcome. Moreover, only 11% of patients in the CS arm were hospitalized for pulmonary edema leading to AVR, and most underwent intervention for mild or moderate symptom onset or anxiety [72].

### 6.2. Hospitalizations and Unplanned Cardiovascular Admissions

Concerning heart failure hospitalizations and unplanned cardiovascular admissions, a meta-analysis by Genereux combining four randomized trials reported a pooled HR of 0.62 (95% CI, 0.40–0.97), indicating a significant reduction with early AVR compared to clinical surveillance. The individual trials showed the following HRs: RECOVERY 0.05 (95% CI, 0.00–1.05), AVATAR 0.21 (95% CI, 0.06–0.73), EVOLVED 0.37 (95% CI, 0.16–0.88), and EARLY TAVR 0.43 (95% CI, 0.33–0.55). Notably, EARLY TAVR carried the greatest weight in this analysis (60.2%) [68,73]. However, this trial included patients with mild symptoms and classified valve replacement after crossover from clinical surveillance as an unplanned hospitalization, which may have biased hospitalization rates in the control group [74]. Nevertheless, these findings collectively highlight the important clinical benefit of early intervention in reducing hospitalizations, likely through relief of left ventricular pressure overload in severe aortic stenosis, thereby improving cardiac function and delaying symptom onset.

### 6.3. TAVR Complications and Safety Profile

An important aspect in evaluating the risk-benefit balance of early intervention is the assessment of procedure-related complications such as stroke, major vascular complications, and bleeding. Notably, the EARLY TAVR trial reported a reduction in stroke incidence in the treatment group. It has been speculated that this finding may be explained by the fact that valvular disease itself causes structural changes in the atrium and ventricle, which can promote the development of subclinical atrial fibrillation, thereby increasing stroke risk. However, this theory needs to be validated. In addition, current-generation TAVR platforms are associated with a low incidence of disabling periprocedural stroke, approximately 1% to 2% in real-world registries, highlighting the improved safety profile of these interventions.

### 6.4. TAVR Long-Term Complications and Follow-Up Considerations

Structural valve deterioration (SVD) has traditionally been defined by the Society of Thoracic Surgeons as the need for aortic valve reintervention or valve-related death but this definition underestimated its true incidence, especially in elderly or high-risk patients [75,76]. To address this limitation, the European Association of Percutaneous Cardiovascular Interventions (EAPCI), the European Association for Cardio-Thoracic Surgery (EACTS), and the Valve Academic Research Consortium 3 (VARC-3) have introduced standardized definitions based on Doppler echocardiographic parameters, allowing for earlier identification and grading of both structural and hemodynamic valve deterioration (HVD) [77]. Bioprosthetic Valve Failure (BVF), which included valve-related death, reintervention, and severe dysfunction, emerged as the most comprehensive measure of valve durability. Recent studies have provided more granular insight into the true incidence and progression of valve degeneration [78]. According to the EAPCI/EACTS definitions, the incidence of moderate or severe (Stage 2 or 3) SVD ranged from 4.6% to 14.9% at 7 to 8 years. Using the VARC-3 criteria, the incidence reached up to 13.9% at 10 years. When including all causes of BVF, such as prosthesis–patient mismatch (PPM), paravalvular regurgitation, thrombosis, or infective endocarditis, the incidence was 9.7% at 10 years [78]. Non-structural valve dysfunction (non-SVD), including severe PPM or paravalvular regurgitation, can negatively impact leaflet motion and transvalvular flow, increasing the risk of thrombotic events and subsequent structural degeneration. Subclinical leaflet thrombosis as hypo-attenuated leaflet thickening (HALT) was observed in 15–32% of TAVI patients within two years and was thought to contribute to valve degeneration. Endocarditis was also shown to accelerate SVD [78]. These observations highlight the complex interplay between structural and non-structural factors in the evolution of bioprosthetic valve dysfunction (BVD) and BVF, underscoring the importance of systematic long-term surveillance. Midterm data comparing TAVR and SAVR using VARC-3 criteria suggest that TAVR prostheses offer durability comparable to surgical bioprostheses [79,80,81,82]. Blackman et al. evaluated long-term valve durability beyond 5 years, with echocardiographic follow-up available in 241 patients at 5 years, but fewer than 15% followed beyond 8 years [83]. Due to the limited long-term data and the older patient population, the results could not be generalized to younger patients, for whom durability is a critical issue. At 10 years, the NOTION trial found similar SVD rates in both groups (12.5% in TAVR and 13.9% in SAVR) [84]. However, interpretation of these long-term results is limited by survivorship bias, the use of early-generation devices, and lack of echocardiographic core lab adjudication. While these findings are generally reassuring, definitive conclusions about long-term (>10 years) valve durability will require data from ongoing trials in low-risk populations, such as PARTNER 3 and Evolut Low Risk, which are expected to report 10-year outcomes within the next five years. Hong-De Li et al. showed that TAVR provides comparable long-term valve durability and stable hemodynamics over 10 years in patients with both BAV and tricuspid aortic valve morphologies, with low rates of SVD and BVF [85].

An additional issue to consider in the risk–benefit assessment of TAVR is the potential need for permanent pacemaker implantation (PPI). Complete atrioventricular block requiring PPI occurs in approximately 15% of patients within 30 days after the procedure, and its incidence is influenced by several factors, including the depth of valve implantation, the type of prosthesis used, patient anatomy, and the presence of pre-existing conduction disturbances [83,86,87]. PPI has been associated with increased short- and long-term mortality, possibly due to the underlying conduction abnormality or factors related to the device itself [88]. These aspects may significantly impact both the expected improvements in quality of life and the overall cost of the procedure. Moreover, current data are mainly derived from elderly and high-risk patients, and it remains uncertain whether these findings are applicable to younger, lower-risk populations [89].

An important consideration in the risk–benefit evaluation of TAVR is coronary re-access, especially in younger patients. Despite improvements in devices and techniques, challenges with coronary cannulation persist, particularly in patients with longer life expectancy and a growing risk of ischemic events over time. In this context, it is therefore crucial to consider both the incidence of ischemic cardiac events and the success of coronary re-access. Overall, the literature on the true incidence of acute coronary syndrome during long-term follow-up after TAVR in real-world settings remains limited [90,91,92]. Although data are scarce, the OBSERVANT study reported cardiac ischemic events in 8.3% of patients at 5 years, with the incidence increasing over time [92,93,94]. Regarding coronary re-access, the RE-ACCESS study found that unsuccessful coronary cannulation occurred in 7.7% of cases, almost exclusively in patients treated with Evolut valves. This risk was strongly associated with factors such as the use of an Evolut valve, a higher valve-to-sinus of Valsalva ratio, and deeper implantation [95,96].

## 7. Future Perspectives: Towards a Personalized Approach

These findings underscore the importance of **patient selection** and the need to weigh the long-term risks of early intervention against the expected clinical benefit, rather than relying solely on composite endpoints. The decision to undertake aortic valve intervention in an asymptomatic patient requires careful consideration, because the early procedural risks need to be weighed against those associated with progressive and potentially irreversible adverse left ventricular remodeling, heart failure, and death with continued conservative management (Figure 1).

**Figure 2:**
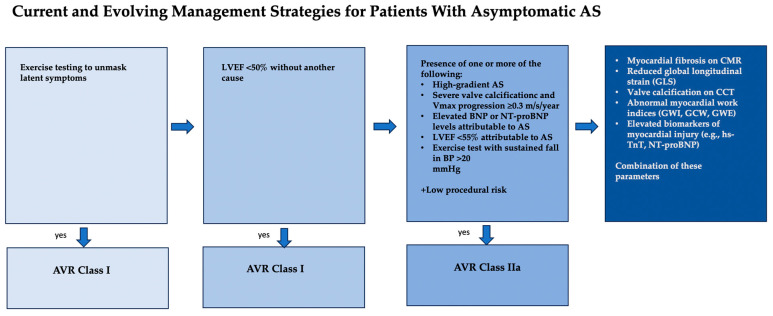
Evaluation and treatment of patients with asymptomatic severe aortic stenosis. Beyond determination of left ventricular ejection fraction and verification of asymptomatic status with exercise testing, there are several established indices of disease severity supported by the American Heart Association/American College of Cardiology (AHA/ACC) and European Society of Cardiology/European Association for Cardio-Thoracic Surgery (ESC/EACTS) guidelines, as well as evolving novel indices. The approach to the asymptomatic patient may include unmasking of symptoms through stress testing, assessment of EF, and evaluation of severity parameters using echocardiography, CT, and stress imaging. Future perspectives involve combining parameters derived from advanced imaging techniques, such as global longitudinal strain (GLS), myocardial work, and myocardial fibrosis on cardiac MRI—with circulating biomarkers, including troponin. AS = aortic stenosis; AVR = aortic valve replacement BNP = B-type Natriuretic Peptide; BP = Blood pressure; CCT = cardiac computed tomography; CMR = cardiac magnetic resonance; hs-TnI = high-sensitivity Troponin I LVEF = left ventricular ejection fraction; GCW = global constructive work; GWI = global work index; GWE = global work efficiency.

The decision-making process for early AVR must carefully balance evolving procedural risks and long-term outcomes. These outcomes have improved significantly due to advancements in valve technology. The use of newer-generation prostheses has resulted in better procedural safety and more favorable long-term results [97,98,99,100,101,102]. Equally important is the enhanced capability to accurately identify truly high-risk asymptomatic patients through advanced screening methods. Inconsistent results across recent trials likely reflect suboptimal patient selection. This underscores the critical need to refine risk stratification. The goal is to avoid overtreatment while ensuring timely therapy for patients at true risk. Patient evaluation should include assessment of aortic stenosis severity both at rest and under stress. Stress echocardiography, in particular, is valuable for evaluating not only symptoms but also hemodynamic gradients. Myocardial damage assessment should extend beyond biomarkers such as high-sensitivity troponin and BNP to include advanced imaging techniques. CRM plays a crucial role by not only identifying the presence or absence of fibrosis but also quantifying fibrosis levels. This quantification helps identify patients at higher risk of adverse outcomes. Also, GLS may play a crucial role in risk stratification. Evaluating GLS both at rest and during stress testing could help define cutoff values for GLS reduction under stress. These cutoffs may identify patients at greater risk. Magne et al. have already demonstrated that patients with an absolute GLS below 14.7% have a 2.5-fold higher risk of death, as shown in a recent individual participant data meta-analysis [48,103] (Figure 2).

Moreover, personalized medicine in the management of asymptomatic severe aortic stenosis should account for sex-related differences in both risk stratification and device selection. Increasing evidence suggests that men and women may exhibit different patterns of disease progression, ventricular remodeling, and response to valve replacement therapies. For instance, women often present with smaller annular dimensions, more concentric hypertrophy, and may be more prone to prosthesis–patient mismatch. These anatomical and physiological differences can influence not only the timing of intervention but also the classification of severity, the choice between surgical and transcatheter approaches, and the type of valve prosthesis. It is also important to note that women have been consistently underrepresented compared to men in the EVOLVED and EARLY TAVR trials (Table 3), which may limit the generalizability of findings across sexes. Sex differences should be considered in risk stratification as well. Recent multicenter studies involving over 600 patients with at least moderate aortic stenosis and preserved ejection fraction have proposed sex-specific thresholds for aortic valve calcium scoring by CT to define severe AS with high accuracy (men: ≥2065 AU or ≥476 AU/cm^2^ indexed to aortic annular area; women: ≥1274 AU or ≥292 AU/cm^2^). This highlights the importance of sex-specific diagnostic criteria in risk assessment, suggesting that different cutoffs may need to be applied [104]. Similarly, sex differences may impact therapeutic approach and device selection [105,106,107]. Recognizing and integrating these sex-specific variations into clinical decision-making is essential to optimize outcomes and ensure equitable care for all patients.

Another subgroup of patients that deserves particular attention in these trials is those with BAV. As expected, the proportion of BAV was lower in the EARLY TAVR study, which evaluated TAVR, compared to trials that investigated SAVR. BAVs present distinct anatomical and pathological complexities that pose significant challenges to the conventional TAVR approach. These differences can impact procedural planning, device selection, and ultimately clinical outcomes. Given the unique nature of BAV, specific strategies and dedicated studies are needed to optimize treatment in this population. The integration of TAV technology in patients with BAV is advancing rapidly in clinical practice. This progress has been supported by careful patient selection, meticulous pre-procedural planning, and specific intra-procedural techniques, particularly in symptomatic patients [2,106,108,109,110,111]. However, dedicated analysis is warranted in asymptomatic patients with severe aortic stenosis and BAV, taking into account their different patterns of disease progression, younger age, and procedural challenges. For this subgroup, a tailored risk–benefit assessment should be considered.

## 8. Conclusions

The management of asymptomatic severe AS remains complex and demands an individualized approach. Decisions regarding early AVR should be based on a careful balance between procedural risks and the potential long-term benefits, which have significantly improved with advances in valve technology. Importantly, despite new evidence available, the 2025 ESC/EACTS guidelines have introduced only minor changes compared to the 2021 guidelines, introducing the indication of AVR for asymptomatic patients with severe, high-gradient AS and LVEF ≥ 50% as an alternative to close active surveillance, if the procedural risk is low and without the need of additional variables (Class IIa, Level of Evidence A). This highlights the ongoing need to precisely identify high-risk individuals who may truly benefit from early treatment, rather than broadening indications indiscriminately. Equally crucial will be the integration of refined risk stratification tools, including advanced imaging techniques, which enhance our ability to detect subclinical myocardial damage and early markers of disease progression. Ultimately, the goal is to avoid unnecessary interventions in low-risk individuals while ensuring timely and appropriate therapy for those at higher risk. This tailored strategy remains essential to optimize clinical outcomes and improve the overall quality of care in patients with asymptomatic severe AS.

## Figures and Tables

**Table 1:** Classification and Management of Symptomatic Aortic Stenosis.

Type of Aortic Stenosis	Diagnostic Criteria	LVEF	Additional Evaluation	AVR Recommendation 2021 ESC/EACT Guidelines	AVR Recommendation 2025 ESC/EACT Guidelines	AVR Recommendation 2020 ACC/AHA Guidelines
**Symptomatic High-Gradient AS**	Symptoms Mean gradient ≥ 40 mmHg Peak velocity ≥ 4.0 m/s AVA ≤ 1.0 cm^2^ or ≤ 0.6 cm^2^/m^2^	Independent of LVEF	Not required	Class I, Level B	Class I, Level B	Class I, Level A
**Symptomatic Low-Flow, Low-Gradient (LFLG) AS with preserved LVEF**	Symptoms AVA < 1.0 cm^2^ Mean gradient < 40 mmHg Peak velocity < 4.0 m/s LVEF > 50% SVi < 35 mL/m^2^	Preserved	DSE CT calcium scoring	Class IIa, Level C (after careful confirmation that AS is severe)	Class IIa, Level B (after careful confirmation that AS is severe)	Class I, Level B (if severity confirmed and symptoms are AS related)
**Low-Flow, Low-Gradient (LFLG) AS with reduced LVEF** **(Classical LFLG)**	Symptoms AVA < 1.0 cm^2^ Mean gradient < 40 mmHg LVEF < 50% SVi < 35 mL/m^2^	Reduced	DSE to assess: - Gradient/velocity - Contractility - AVA - Flow reserve (SV ↑ > 20%) CT calcium score (if no reserve)	Class I, Level B (With flow reserve and AVA < 1.0 cm^2^) but severe AS confirmed by CCT Medical therapy in pseudo AS (With flow reserve and AVA > 1.0 cm)	Class I, Level B (after careful confirmation that AS is severe)	Class I, Level B

DSE = dobutamine stress echocardiography; AS = aortic stenosis; LVEF = left ventricular ejection fraction; CCT = cardiac computed tomography; LFLG = Low-Flow, Low-gradient.

**Table 2:** Studies Comparing Early AVR vs. Clinical Surveillance in asymptomatic severe AS.

Author, Year	Study Type	Number of Patients	AVR Type	Mortality/Follow-Up (AVR vs. CS)
Pai et al., 2006 [59,60]	Retrospective observational	338 asymptomatic patients	SAVR	5-year survival: 90% vs. 38%; HR 0.17 (95% CI 0.10–0.29)
Taniguchi et al., 2015 [60]	Retrospective propensity matched	582 (291 AVR vs. 291 CS)	SAVR	5-year mortality: 15.4% vs. 26.4% (*p* = 0.009)
Kang et al., 2010 [61]	Retrospective propensity matched	114 (57 matched pairs)	SAVR	HR 0.14 (95% CI 0.03–0.60); 6-year survival 98% vs. 68%
AVATAR, 2020 [63]	Randomized controlled trial	157	SAVR	Up to 5 years follow-up; composite outcome significantly better with early AVR
RECOVERY, 2020 [64]	Randomized controlled trial	145	SAVR	Median follow-up 6.2 years; better survival with early AVR
EVOLVED, 2024 [65]	Randomized controlled trial	224	TAVR or SAVR	HR for composite endpoint 0.79 (95% CI 0.44–1.43)
EARLY TAVR, 2024 [66]	Randomized controlled trial	901	TAVR	Median follow-up 3.8 years; mortality 8.4% vs. 9.2%; HR for composite endpoint ~0.50

AVR = Aortic Valve Replacement; CI = Confidence Interval; CS = Clinical Surveillance; HR = Hazard Ratio; *p* = *p*-value; SAVR = Surgical Aortic Valve Replacement; TAVR = Transcatheter Aortic Valve Replacement.

**Table 3:** Randomized Controlled Trial comparison: baseline characteristics.

Author, Trial	Age		Sex, Female		BAV		STS Score EUROSCORE II		AVA, cm^2^		Vmax, m/s		MG, mmHg	
	Early AVR	CS	Early AVR	CS	Early AVR	CS	Early AVR	CS	Early AVR	CS	Early AVR	CS	Early AVR	CS
AVATAR [63]	68 (63–73)	69 (64–75)	32 (41%)	35 (44%)	61%		1.6 (1.1–2.2)	1.8 (1.2–2.7)	0.73 (0.55–0.84)	0.74 (0.59–0.89)	4.5 (4.3–4.8)	4.5 (4.2–4.7)	51 (33–58)	50 (43–59)
RECOVERY [64]	65.0 ± 7.8	63.4 ± 10.7	36 (49%)	38 (53%)	49 (67%)	39 (54%)	0.9 ± 0.3	0.9 ± 0.4	0.63 ± 0.09	0.64 ± 0.09	5.14 ± 0.52	5.04 ± 0.44	64.3 ± 14.4	62.7 ± 12.4
EVOLVED [65]	75 (86–79)	76 (68–80)	31 (27%)	32 (29%)	36 (32%)	28 (25%)			0.8 ± 0.2	0.8 ± 0.2	4.3 ± 0.5	4.4 ± 0.5	45.2 ± 11.5	45.0 ± 10.2
EARLY TAVR [66]	76.0 ± 6.0	75.6 ± 6.0	131 (29%)	147 (33%)	37 (8.1%)	39 (8.8%)	1.8 ± 1.0	1.7 ± 1.0	0.9 ± 0.2	0.8 ± 0.2	4.3 ± 0.5	4.4 ± 0.4	46.5 ± 10.1	47.3 ± 10.6

AVA = Aortic Valve Area; AVR = aortic valve replacement; BAV = Bicuspid Aortic Valve; CS = Clinical Surveillance; EUROSCORE II = European System for Cardiac Operative Risk Evaluation II; MG = Mean Gradient; STS score = Society of Thoracic Surgeons score; Vmax = Peak Velocity.

## Data Availability

No new data were created or analyzed in this study.

## Abbreviations

The following abbreviations are used in this manuscript:

AS	Aortic stenosis
AVA	Aortic valve area
AVR	Aortic valve replacement
BAV	Bicuspid aortic valve
CCT	Cardiac computed tomography
CMR	Cardiac magnetic resonance
CS	Clinical surveillance
GLS	Global longitudinal strain
LVEF	Left ventricular ejection fraction
SAVR	Surgical aortic valve replacement
TAVR	Transcatheter aortic valve replacement
